# *Corynebacterium bovis* Eye Infections, Washington, USA, 2013

**DOI:** 10.3201/eid2109.150520

**Published:** 2015-09

**Authors:** Siu-Kei Chow, Uyen Bui, Jill E. Clarridge

**Affiliations:** University of Washington, Seattle, Washington, USA (S.-K. Chow, J.E. Clarridge);; Veterans Administration Puget Sound Health Care System, Seattle (U. Bui, J.E. Clarridge)

**Keywords:** eye infections, ophthalmology, *Corynebacterium*, veterinary, bacteria, Washington, United States, zoonoses

**To the Editor:** *Corynebacterium bovis* is well known as a normal bovine microbiota and is a common cause of bovine mastitis (*1*). *C. bovis* infections in humans are rare, and identification of the organism by biochemical methods is challenging (*2*). Although 9 cases of *C. bovis* infections in humans have been reported (*3**–**6*), only the most recent case, which involved prosthetic joint infection, used 16S rRNA gene sequencing to identify the bacterium with certainty (*6*).

During February*–*July 2013, four adult patients (Table) were seen at Veterans Administration Puget Sound Health Care System in Seattle, Washington, USA, for eye swelling, pain, and purulent discharge. All 4 cases were associated with isolation of *C. bovis* from essentially pure culture. We investigated these 4 cases after obtaining approval from the Puget Sound Veterans Administration Medical Center Institutional Review Board (MIRB #01012).

**Table:** Characteristics and test results for 4 isolates of *Corynebacterium bovis* from patients with eye infections, Washington, USA, 2013*

Characteristic						Reference strains		
						*C. bovis* type strain ATCC 7751	Animal isolates,† n = 115	Human isolates,† n = 6
	Isolates from this study							
	F7181	F7545	F7275	F7551				
Patient no.	1	2	3	4				
Age, y/sex	49/M	25/M	33/M	90/M				
Date isolated	2013 Feb 4	2013 Jul 2	2013 Feb 27	2013 Jul 12				
Location where specimen was collected	VAPSHCS urgent care	VAPSHCS urgent care	VAPSHCS ED	Bellevue clinic				
API Coryne test code	2001004	0001004	0001105	0001004				
Test results								
Production of								
Catalase	+	+	+	+		+	100	100
Urease	+	+	+	+		–	44–68	17
Pyrazinamidase	+	–	–	–		+	ND	ND
Acid production from								
Glucose	–	–	+	–		+	56–98	50
Galactose	–	–	–	–		+	11–31	33
Mannose	–	–	–	–		+	5–33	33
Lactose	–	–	–	–		+	11–26	17
Sucrose	–	–	+	–		ND	ND	ND
GenBank accession no.	KJ769199	KJ769200	KJ769201	KJ769202				
16S rRNA gene sequence identity to type strain ATCC 7751, %	100	100	100	100				
Length of 16S rRNA gene sequence, nt	465	424	465	436				

*Phenotypic characteristics of *C. bovis* isolates from this study (*2*) were obtained by using API Coryne system v3.0 and Vitek2 (bioMérieux, Marcy  l’Etoile, France). +, positive; –, negative; ED, emergency department; ND, not determined; VAPSHCS, Veterans Administration Puget Sound Health  Care System. †% strains positive.

Patient 1 was a 49-year-old man with swelling of the right eyelid with discharge and pain after an episode of itching. Before this visit, the patient had 3 similar episodes and received incision and drainage of the eyelid. On examination, the inverted lower palpebrum revealed a purulent cyst (diameter 1*–*2 mm); pus was collected from the cyst for culture. The patient was prescribed tobramycin ophthalmic drops and amoxicillin/clavulanic acid. No follow-up information was available.

Patient 2 was a 25-year-old man with bilateral eye infection that started on the left eye a week before the patient sought care. The eye had redness, swelling, blurred vision with loss of acuity, and irritation. The right eye had the same symptoms on the day of visit. Examination found bilateral keratoconjunctivitis and a 3-mm cyst with drainage on the lower palpebrum. The patient was treated with ofloxacin ophthalmic drops for 4 months but did not improve. A specimen was then collected from his right eye for culture. In 2014, he was given a diagnosis of chronic conjunctivitis.

Patient 3 was a 33-year-old man with severe pain, erythema, and swelling on his left eyelid. His symptoms started 1 week before he sought care and included swelling, increased cyst size, and disturbed vision. The pustular exudate was aspirated and sent to the laboratory. The patient was prescribed erythromycin ointment and oral trimethoprim/sulfamethoxazole. The patient’s eye had improved at 3 weeks.

Patient 4 was a 90-year-old man who fell at home 2 days before his visit. He landed on his cheek, causing an abrasion, and his eye was swollen shut a few hours after the fall. On the second day, his cheek was swollen and reddened, and yellowish purulent matter was present on the skin. A swab specimen was collected from the wound and sent for culture. The patient was treated with doxycycline for 14 days, and the wound healed by day 12.

The aerobic cultures of 3 eye and 1 cheek wound specimens from these patients grew gram-positive bacilli (Table). The organism was initially identified by the API Coryne system (biomérieux, Marcy l’Etoile, France) as *C. urealyticum* or *Corynebacterium* group F-1. However, given the difficulty of phenotypic identification and the lack of literature to support eye infections associated with *C. urealyticum*, we performed 16S rRNA gene sequence analysis of the first ≈500 bp to confirm the identity. Using the MicroSeq 500 database version 0023b (Applied Biosystems, Foster City, CA, USA), we identified all 4 isolates as *C. bovis* (100% identical to ATCC 7715; sequence length 424–465 nt). According to the MicroSeq 500 and GenBank databases, the next 2 closest matches were *C. confusum* (96.1% similarity) and *C. macginleyi* (95.9% similarity), making the identification unambiguous.

*C. bovis* has not been described as part of the human microbiota, nor has it been associated with eye infections, in contrast to other *Corynebacterium* spp. known to colonize the human conjunctiva and skin (*7*) and cause eye infections (*8**,**9*). We found *C. bovis* associated with each of these eye and facial soft-tissue infections, but whether this lipophilic organism colonizes in the oily glands of eyelids in healthy individuals is unclear. What is certain is that *C. bovis* can exist on the human facial skin, has pathogenic potential, and is difficult to identify.

Because human and animal strains of *C. bovis* vary in biochemical properties (*2*), phenotypic identification is unreliable. All our isolates were urease positive, contrary to most isolates reported in the literature (*2*). This phenotype may result in underreporting of the organism because it is not described in some databases (*10*). An epidemiologic investigation revealed no overlap among any of the specimens regarding date of collection, clinic location where patients were seen, or date of clinical work-up. From results of our investigation, we believe that cross-contamination was unlikely and that these cases are probably independent of each other.

The pathogenicity of *Corynebacterium* spp. can be easily overlooked, especially because some species are common skin colonizers. Speciation should be prompted when *Corynebacterium* spp. are isolated in large quantity or from a pure culture. Unexpected phenotypic identifications such as *C. urealyticum* from eye specimens should be confirmed with 16S rRNA gene sequencing.
